# A New Insight into Toxicity of Colchicine Analogues by Molecular Docking Analysis Based on Intestinal Tight Junction Protein ZO-1

**DOI:** 10.3390/molecules27061797

**Published:** 2022-03-09

**Authors:** Jiali Liu, Rongrong Gao, Xuejing Gu, Bin Yu, Yan Wu, Qiushi Li, Ping Xiang, Hui Xu

**Affiliations:** 1 School of Pharmacy, Key Laboratory of Molecular Pharmacology and Drug Evaluation (Yantai University), Ministry of Education, Collaborative Innovation Center of Advanced Drug Delivery System and Biotech Drugs in Universities of Shandong, Yantai University, Yantai 264000, China; 17806097660@163.com (J.L.); 17862191758@163.com (R.G.); guxuejingll@163.com (X.G.); medicine2134@163.com (B.Y.); wuyan662185@163.com (Y.W.); liqiushi2018@163.com (Q.L.); 2 Shanghai Key Laboratory of Forensic Medicine, Shanghai Forensic Science Platform, Academy of Forensic Science, Ministry of Justice, Shanghai 200063, China

**Keywords:** colchicine analogues, gastrointestinal toxicity, intestinal tight junction protein ZO-1, molecular docking, structure–toxicity relationship, metabolic detoxication

## Abstract

Colchicine (COL) is a well-known plant alkaloid long used for medical purposes due to the selective anti-inflammatory effect on acute gouty arthritis. It is also a kind of mitosis toxin with strong inhibitory effects of cell division and is therefore being applied to the treatment of various cancers. However, this product shows a variety of adverse effects that are significantly correlated with the dosage and have attracted much attention. For the first time, the present work obtained a new insight into the gastrointestinal toxicity of colchicine analogues by molecular docking analysis, which was based on the 3D structure of intestinal tight junction protein ZO-1 and the ligand library containing dozens of small-molecule compounds with the basic skeleton of COL and its metabolites. The binding energy and mode of protein–ligand interaction were investigated to better understand the structure–toxicity relationships of COL analogues and the mechanism of action as well. Cluster analysis clearly demonstrated the strong correlation between the binding energy and toxicity of ligand molecules. The interaction mode further revealed that the hydrogen bonding (via the C-7 amide or C-9 carbonyl group) and hydrophobic effect (at ring A or C) were both responsible for ZO-1-related gastrointestinal toxicity of COL analogues, while metabolic transformation via phase I and/or phase II reaction would significantly attenuate the gastrointestinal toxicity of colchicine, indicating an effective detoxication pathway through metabolism.

## 1. Introduction

The gut barrier is the largest barrier in vivo, separating the internal and external environment, can protect the body from hazardous and toxic substances outside [1]. Tight junction proteins (TJP) are the main proteins that maintain the tight junctions (TJs) of intestinal epithelial cells and play a vital role in cell differentiation. Zonula occludens-1 (ZO-1) is one of the important proteins of TJP, which is indispensable in the penetration between adjacent cells, signal transduction, gene transcription, cell proliferation and differentiation [2]. Since the structure and function of ZO-1 are closely correlated with other members of TJs, functions of TJs will change with the destruction of ZO-1 in most cases. Therefore, ZO-1 is always used as an index and marker to observe various tissues’ barrier function and permeability function [3,4].

Colchicine (COL, 1) is a natural alkaloid of tropolone derived from the lily family *Colchicum autumnale*. It is one of the oldest natural plant products and has been used as a traditional medicine for around 2000 years [5,6]. As a typical natural mitotic toxin, COL has functioned with anti-inflammatory and pharmacological activities of fibroblast proliferation, showing more surprising clinical applications in treating immune system diseases such as gout, cancer, familial Mediterranean fever, and Behcet’s disease [7,8]. Although it has significant efficacy in treatment, the narrow therapeutic window of COL is still an obstacle to its clinical application [9]. Colchicine itself is too potent to use in chemotherapy, and even for gout treatment, doses of colchicine must be strictly administered to avoid toxicity [10]. According to reports, in the clinical application of COL, there is a general safety risk that the toxic dose is very close to the safe dose. At the same time, it is widely distributed in vivo and has local reactions [11]. The toxicokinetic studies showed that improper use of COL could cause serious adverse reactions, such as intestinal damage, liver toxicity, acute renal failure, and hematological toxicity [12]. The difficulty in managing the toxicity of COL is that preventive strategies and precise mechanisms for the toxic effects of COL are still elusive. With the COL applied rapidly in clinical treatment, concerns about its potential toxicity are increasing.

Adverse gastrointestinal reactions are the key biological events of COL poisoning [13]. Acute oral administration can influence the structure of the gastrointestinal tract significantly and significantly transform the composition, function, and diversity of the intestinal microbiome. Animal studies have shown that COL could break the integrity of the intestinal barrier by destroying the microtubule cytoskeleton, resulting in increased intestinal permeability, leading to more severe intestinal damage and diseases, such as inflammatory bowel disease, irritable bowel syndrome, colon cancer, and so on [10,14]. Moreover, COL could inhibit cell growth and induce apoptosis of intestinal crypt cells. Existing studies that had taken immunohistochemical staining verified that COL could enhance intestinal permeability by reducing the expression of ZO-1 protein to trigger endotoxemia in animals [13]. Therefore, we have noted a characteristic and distinctive type of mucosal injury of the gastrointestinal tract and its association with clinical COL toxicity. It is a possible way to study and elaborate the mechanism of COL, inducing adverse gastrointestinal reactions from the perspective of ZO-1 [14].

Molecular docking is a kind of technology using receptors and ligands with known structures to recognize and predict the structure of receptor–ligand complexes based on the principles of energy and chemical environment complementation. It can simulate drug–receptor interactions, clarify the mechanism of drug action, and improve the accuracy, sensitivity, and predictability of drugs, making it a valuable tool for drug development [15]. Molecular mechanics with generalized Born and surface area solvation (MM/GBSA) is a method to calculate the binding energy between two molecules based on molecular dynamics and continuum model. This method could achieve a good balance between computational efficiency and accuracy and increase molecular docking reliability [16]. MM/GBSA has been widely used to evaluate docking poses, determine structural stability, and predict binding affinities and hotspots, providing new means to explore the action mechanism of small molecular drugs [17].

**Figure 1:**
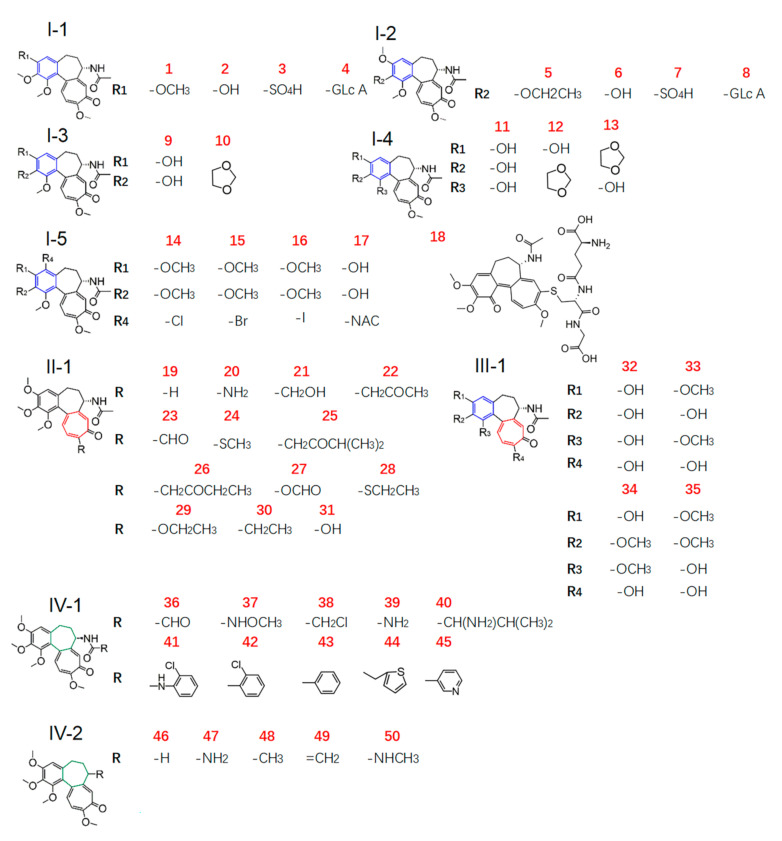
Chemical structures (type I to IV) of COL analogues in the ligand library.

In the present work, we aimed to obtain new insight into the gastrointestinal toxicity of colchicine analogues by molecular docking analysis. The human ZO-1 was used as the target protein, and the ligand library was composed of dozens of small-molecule compounds, including a total of 50 COL analogues with the basic skeleton of COL and its metabolites (Figure 1 and Table S2), some protective agents, and those with intestinal injury effect (Table 1) used as a control for comparison. The binding energy and mode of protein–ligand interaction were investigated better to understand the structure–toxicity relationships of these COL analogues and the mechanism of action.

## 2. Results and Discussion

### 2.1. Molecular Docking and Cluster Analysis

**Figure 2:**
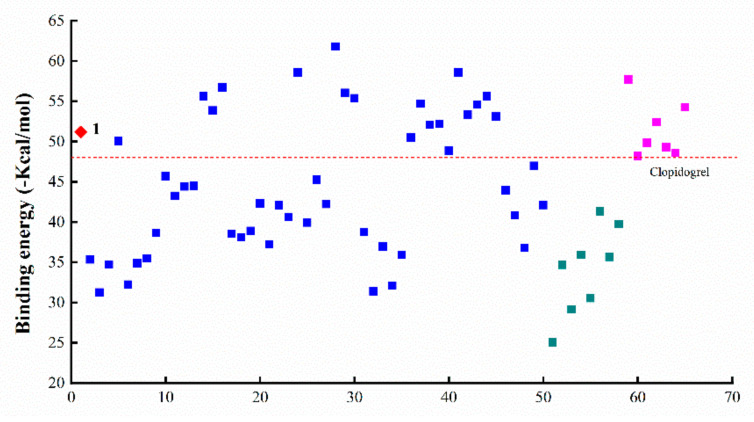
Binding energy of the interactions between ZO-1 and miscellaneous ligands (◆ colchicine, ■ colchicine analogues, ■ intestinal injury compounds, ■ intestinal protective compounds).

By using the MM/GBSA method, molecular docking was performed for a total of 65 small-molecule compounds in the ligand library (Figure 1 and Table 1) and the binding energy (BE) of ligand–protein interaction was calculated for a quantitative appraisal (the detailed free energy information of the compounds was shown in Table S1). Consequently, the ligands all displayed a minus value of BE with the absolute ranging from 25–65 (Figure 2), suggesting the formation of a stable ligand–protein complex and the obvious variation among all the ligands. When compared with the reference ligand clopidogrel (60) that had a BE value of 48.08 kcal/mol along with an obvious injury effect as previously reported [18], there were 25 compounds that exhibited a higher absolute value of BE, including COL and all other intestinal injury agents. Even more interesting was that the ligands with lower total BE value involved those injury protectors and all the metabolites of COL.

**Figure 3:**
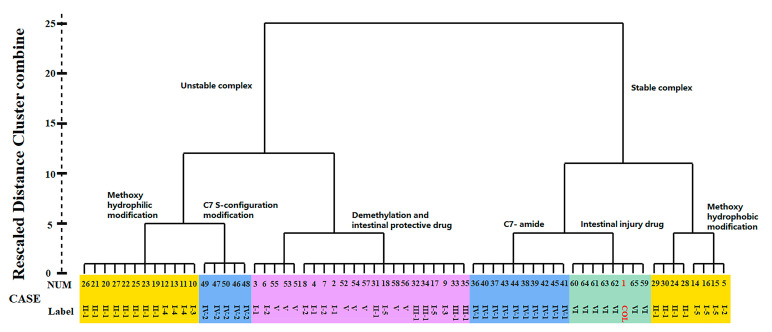
Dendrogram of clustering analysis according to the BE value of miscellaneous ligands.

The cluster analysis was further performed according to the BE values of miscellaneous compounds in the ligand library, which contained not only COL analogues (**1**–**50**, Type I–IV) but also some protectors (**51**–**58**) and those with intestinal injury potential (**59**–**65**, Type VI) as a control for comparison. A dendrogram was obtained with good discrimination for the two control groups with different activities (Figure 3). More to the point, it was found that those intestinal injury drugs (Type VI) along with COL were clustered into the same class with fairly high BE values. Therefore, these findings clearly demonstrated the strong correlation between the potency of ZO-1 related activities and the BE values of ligand-protein interaction. Specifically, the absolute value of BE seemed to be positively correlated with the injury effect of ligand, thus could be used as a reliable indicator to measure ZO-1-related intestinal injury toxicity for future studies on the structure–toxicity relationship.

### 2.2. Structure–Toxicity Relationship of COL Analogues

#### 2.2.1. An Overview Based on Cluster Analysis

COL is a natural alkaloid with a 6/7/7 tricyclic ring system, including a tropolone ring and a steric center at the C-7 position leading to an *S*-configuration of the chiral axis defined by the bond joining rings A and C [19,20,21]. A variety of studies have demonstrated the influence of structural modification of COL on its therapeutic effects [8,22,23]. It has also been found that the methoxyl groups on rings A and C are essential to maintain the cytotoxicity of COL. In contrast, modifications on ring B have little influence on its activities [8].

The present study investigated the relationship between chemical structure and ZO-1-related toxicity of COL analogues according to cluster analysis of the BE values. As illustrated in Figure 3, all the ligand library compounds were first clustered into two groups with stable and unstable bonds, respectively. Furthermore, the group with stable bonds could be well divided into three subgroups, including the analogues with C-7 amido modification, those with modification of methoxyl on ring A/C into hydrophobic groups, and the positive control drugs intestinal injury potential (type VI). According to the variation in modification, the group with unstable bonds was also divided into several subgroups, including the analogues with modification of methoxyl on ring A/C into hydrophilic groups, chiral modification of ring B, and those protectors from intestinal injury. These results thus indicated a close relationship between chemical structure and ZO-1 binding affinity, which may greatly impact the intestinal injury-related toxicity of COL analogues.

#### 2.2.2. Effect of Modification on Ring A

Ring A of COL is a benzene ring containing three substituents. For COL itself, the changes in three methoxyl groups usually lead to the loss of activity. It has been recently reported that demethylation of one or more methoxyl groups may decrease anti-mitotic activity by several orders of magnitude [24]. Herein, there was a total of 21 COL with modification at ring A (**2**–**17**, **32**–**35**), including demethylation, methylenedioxy bridge, O-glycoside residue substitution, etc. Consequently, there were significant differences in the interaction with the target protein ZO-1 among these ligands (Figure 2), suggesting the great impact of the substituent on ring A. More to the point, the four compounds with BE values comparable to COL and higher than the reference ligand were **5**, and **14**, **15**, **16** which had a structure of type I-5 with halogen substituent at C-4 as shown in Figure 1. This result demonstrated that the interaction between COL and ZO-1 would be little affected by mono-halogenation at C-4 or solely replacing the methyl part of the methoxyl group with alkyl, which thus was not the key role affecting the intestinal injury toxicity of COL.

In contrast, all the other 17 analogues showed a much lower absolute value of BE than COL. Chemically, they were all found as the demethylation derivatives except analogue **10** with a structure of type I-3 as shown in Figure 1, which was a kind of colchicum alkaloid, namely cornigerine, derived from COL by replacing the methoxyl groups at C-2/C-3 with a methylenedioxy bridge to form a dioxol ring. Moreover, **32**, a COL analogue derived from simultaneous demethylation at C-1, C-2, C-3, and C-10, had the lowest BE value (31.4 kcal/mol) among these ligands. These findings together, therefore, indicated that the methoxyl groups on ring A may be essential for ZO-1 related intestinal injury toxicity of COL analogues, and demethylation reactions would weaken their ZO-1 binding ability, thus leading to toxicity reduction.

#### 2.2.3. Effect of Modification on Ring B

COL has a stereocenter at C-7 on the seven-membered ring B, inducing an S-configuration of the chiral axis defined by the bond joining rings A and C; thus, various analogues could be obtained by replacing the acetamide moiety at C-7 with other amide groups [21]. In order to investigate how the modification on ring B would affect ZO-1 related intestinal injury toxicity of COL, a total of 15 derivatives were thus involved in the present study of the structure–toxicity relationship through molecular docking analysis. Chemically, these analogues belong in two groups with the structure of type IV-1 and IV-2 (Figure 1), which were obtained by replacing the methyl group in amide by various amine groups and aromatic ring such as benzene and thiophene (**36**–**45**), and the compounds no longer with a stereocenter at C-7 (**46**–**50**), respectively.

As a result, the two groups exhibited significantly different BE values of ligand–protein interaction and belonged to different clusters according to further hierarchical clustering analysis (Figure 3). More to the point, the analogues **36**–**45** with type IV-1 structure all had a BE value higher than the reference and comparable to COL, while those in the other group showed an opposite pattern with the BE values all below the reference line (Figure 2). Thus, it was suggested that the integrity structure of amide at C-7, together with the pre-organized chiral configuration, may have a great impact on COL analogues interacting with ZO-1 and the relevant activities. Chemically, the amide group could act as a hydrogen bond donor to form a thermodynamically stable ligand–protein complex with a fairly high BE value similar to COL. In fact, demecolcine (**50**) is a kind of cell-cycle-specific agent from decarbonylated modification of COL and has been widely used for cancer therapy with significantly reduced toxicity [25], thus providing evidence to confirm the findings from the present in silico study on the structure–toxicity relationship of COL analogues. In short, chemical modifications on ring B would not significantly affect the activity of COL analogues unless the amide structure or the stereocenter at C-7 was changed. Such modifications may provide a feasible way to reduce the toxicity and side effects of COL.

#### 2.2.4. Effect of Modification on Ring C

COL is a proto-alkaloid or biological amine with a characteristic tropolone ring C. This moiety has been demonstrated to be responsible for its various activities such as anti-inflammation, antibacterial, and anti-tumor effects [25,26]. As discussed above, the planar structure of tropolone ring C and the hydrophobic methoxyl at C-10 greatly impacted COL analogues’ activities by affecting their interaction with the relevant protein. Herein, a total of 13 COL analogues (**19**–**31**) were involved in molecular docking analysis to understand better how the modification on ring C would affect ZO-1-related intestinal injury toxicity of COL. Chemically, these ligands with a type II structure, as shown in Figure 1, were all obtained from chemical modification at C-10.

According to the docking analysis, all these ligands could be divided into two groups that exhibited significantly different BE values of ligand–protein interaction. One group consisted of four ligands **24** and **28**–**30**, which had hydrophobic substituents such as aliphatic groups, and all displayed a BE value much higher than COL. In particular, the analogue **28** (thiocolchicine) with a substituent group of ethylthio at C-10 exhibited the highest absolute value of BE (−61.79 kcal/mol), suggesting the formation of stable complexes, and also the improved activities related with ZO-1. The clustering analysis further illustrated that these ligands were clustered very close to COL and those positive agents with intestinal injury effect (Figure 3).

In contrast, the other 9 analogues (**19**–**23**, **25**–**27**, and **31**) showed an opposite pattern with BE values all below the reference line (Figure 2), which chemically contained hydrophilic substituents such as aldehyde, ketone, and amine groups at C-10. Therefore, these results demonstrated that ZO-1 related activities of COL analogues might be greatly affected by the substituent groups at C-10 on tropolone ring C. The hydrophilic substituents at C-10 seem to be a sustainable alternative to reduce the toxicity of COL. At the same time, the substitution with hydrophobic groups could maintain or even enhance the relevant activities of COL.

### 2.3. Effect of Metabolic Transformation

Toxicokinetic studies have shown that COL is readily absorbed from the gastrointestinal tract following a rapid metabolic transformation in the liver. Consequently, up to 40% of the drug is excreted in the urine, among which the unchanged drug accounts for only 20–30% [27]. It has also been found that cytochrome P450 (CYP) 3A4 plays a pivotal role in governing metabolic disposition of COL in vivo through the phase I re-action, producing several demethylated derivatives such as 2-DMC, 3-DMC, and 10-DMC [28,29]. These demethylated compounds are prone to further phase II conjugation with hydrophilic moieties (i.e., glucuronate, sulfate, and glutathione) to form more readily excreted metabolites [30,31]. A variety of studies have also demonstrated the great changes in bioactivities of COL caused by metabolic transformation [32]. For example, COL can induce a sharp increase in the level of serum transaminases such as AST and ALT, on which the demethylated metabolites 2/3/10-DMC under the same conditions have little influence, suggesting reduced liver toxicity of COL along with metabolism [12]. However, it is not clear yet how the metabolic reaction would affect ZO-1-related intestinal injury toxicity of COL.

**Figure 4:**
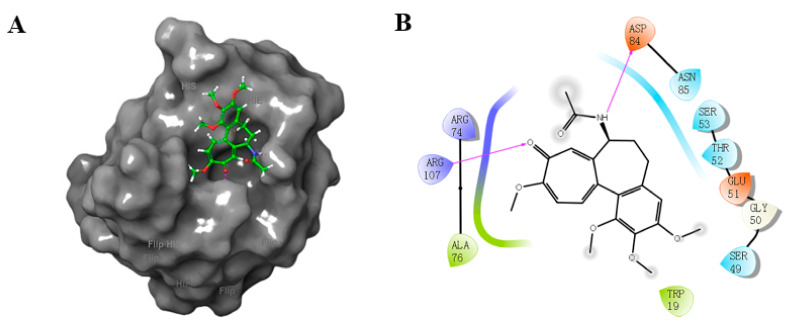
3D (**A**) and 2D (**B**) diagram illustrating interaction between ZO-1 and COL. Gray region: ZO-1, green region: COL. Purple arrow: hydrogen bond, purple: charged (positive), green: hydrophobic, blue: polar, white: glycine.

**Figure 5:**
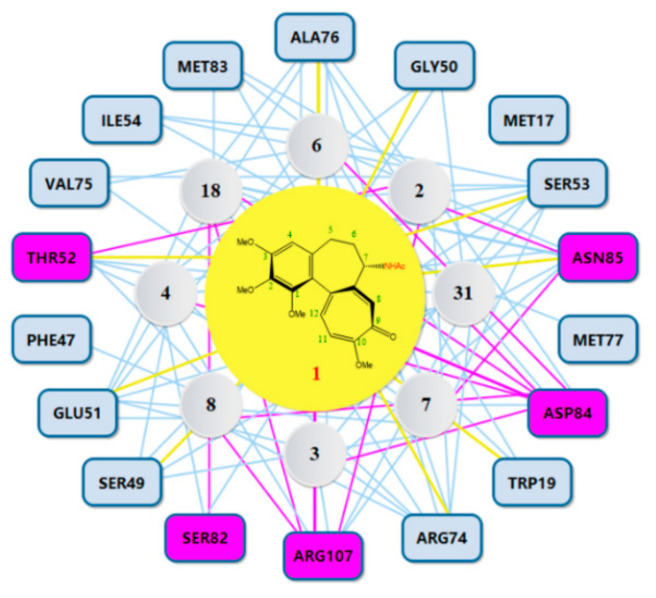
Network diagram illustrating the principal amino acid residues involved in interactions between COL and its metabolites and ZO-1. Purple rectangles represent residue involved in hydrogen bonding. Yellow lines represent residues interacting with COL.

Our present study involved for the first time eight major metabolites of COL from phase I and/or phase II reaction (**2**–**4**, **6**–**8**, **18**, **31**, Figure 1) in the in silico molecular docking analysis. The BE value and interaction mode were investigated to reveal the material basis and possible mechanism of action associated with COL in vivo by using the parent compound as a control for comparison. Resultantly, the parent COL was found to be tightly bound to the target protein ZO-1 via hydrogen bonding, hydrophobic interactions, and electrostatic attraction (Figure 4A,B). For all these ligands, including COL and its metabolites, the network diagram (Figure 5) further illustrates the principal amino acid residues involved in their interactions with the protein ZO-1, while the major statistics of docking analysis are summarized in Table 2.

Firstly, it was found that these ligands all displayed a fairly large negative value of the binding energy that ranged from −31 to −51 kJ/mol, suggesting the formation of a stable complex of ZO-1 with COL or its metabolites. The results of interaction mode analysis further revealed that the protein ZO-1 interacted with these COL analogues mainly through hydrogen bonding, and the residues involved were Trp19, Ser49, Gly50, Glu51, Thr52, Ser53, Arg74, Ala 76, Asp84, Asn85, and Arg107 for COL, and Ser53, Arg74, Ala76, Asn85, Asp84, and Arg107 for its metabolites, respectively (Table 2 and Figure 5). Thus, it was demonstrated that the C-9 carbonyl as a hydrogen bond acceptor, together with the C-7 amido as a hydrogen bond donor and the hydrophobic center, may be greatly responsible for the stable complexing of these COL analogues with the protein ZO-1.

On the other hand, the metabolites all exhibited much lower BE values than the parent COL, suggesting a great attenuation of the ZO-1-related intestinal injury toxicity of COL caused by phase I or phase II metabolic transformation. More to the point, there was no significant difference in the BE values among these metabolites, although they had different interaction forces and the hydrogen bonding sites for complexing. These results provided additional evidence for the structure–toxicity relationship of COL analogues from a new viewpoint and were in good agreement with those cell/animal-based experimental observations previously reported [12,32]. Further, considering the fact that COL in vivo is apt to form more readily excreted metabolites, the findings from the present study thus clearly demonstrated that metabolic transformation through phase I demethylation and phase II conjugation with hydrophilic moieties would lead to in vivo detoxification for COL.

## 3. Materials and Methods

### 3.1. Software and Platform

The software and platform for molecular docking analysis were composed of the Schrödinger software suite (Maestro, v11.8, LLC, New York, NY, USA, 2018), the RCSB Protein Data Bank (http://www.rcsb.org/, accessed on 23 December 2021), the protein database Uniprot (http://www.rcsb.org/, accessed on 23 December 2021), the target and biological activity database EBI (https://www.ebi.ac.uk/chembl/, accessed on 23 December 2021), and ZINC database (https://zinc20.docking.org/, accessed on 23 December 2021).

### 3.2. Preparation and Optimization of the Target Protein

The crystal structure of ZO-1 (PDB ID: 4OEO) with a resolution of 1.9 Å [33] was downloaded from the RCSB protein database then imported into Maestro for pre-treatment using the protein preparation wizard function. In brief, the protein preparation involved the following steps such as adding the missing hydrogen atoms, side chains, and rings, depleting water molecules away from non-standard residues and providing coordinate ions, specifying the bond and metal orders, optimizing the hydrogen bond network via H-bond assignment section, and predicting the optimal protonation state of related amino acid residues under a pH value of 7.0 ± 2.0. Finally, the target protein was obtained with the least energy through the OPLS2005 force field to realize the optimization and perfection of the crystal form.

### 3.3. Construction and Optimization of the Ligand Library

The ligand library for docking analysis consisted of 65 small-molecule compounds with miscellaneous structures. One part was a total of 50 tropolonoids, including the parent COL and its metabolites (Figure 1). These COL analogues with the basic skeleton of colchicine were found by a retrieval using keywords of “colchicine” “colchicine derivative” or “colchicine modification” from the ZINC database and relevant literature. Meanwhile, seven intestinal injury agents and eight protectors were collected to control and compare, and the details are shown in Table 1.

The 2D structure pattern of each ligand was obtained first by the 2D Sketcher function in the Maestro graphical user interface, then edited and converted to a 3D structure for saving. Then, the ligand was assigned the OPLS2005 force field using the LigGrep function, followed by protonation at pH 7.0 ± 2.0 under the Epik mode to obtain the lowest potential energy conformation. The optimized result was saved in .maegz format and used as a starting point for further docking experiments.

### 3.4. Selection of Target Protein Binding Site

The binding pockets of the target protein ZO-1 are not clear yet. The Sitemap pro-gram in the Schrödinger software suite was performed to search for potential active sites since it has become an effective means to identify and characterize binding sites with high accuracy (>96%) [34,35]. The possibility of site determination depended on the site’s proximity to the protein and the degree of solvent protection, and those sites leading to the tightest ligand–protein or protein–protein complex were selected.

### 3.5. Molecular Docking Analysis and Data Mining

The Glide module performed molecular docking analysis in the Schrödinger soft-ware suite and the standard precision (SP) mode was used with the flexible ligand and rigid protein. The optimal ligand conformation and active binding sites were screened according to the GlideScore obtained from Emodel scoring function [36]. Then, the active sites were defined by using the “receptor grid generation” module and located into the domain of the corresponding ligand to generate a network around it, where the van der Waals radius scaling factor was set at 1.0 and the partial charge cut-off value was set at 0.25.

Further, the generalized Born and surface area continuum solvation (MM/GBSA) module was used to obtain binding energy (BE) of the ligand–protein complex for quantitative evaluation of the binding affinity from SP docking [37]. Based on the BE values of the ligand–protein complex, the clustering for data mining was performed by a hierarchical cluster analysis method in SPSS 20.0 (International Business Machines Corporation, New York, NY, USA).

## 4. Conclusions

In conclusion, the present work got a new insight into the gastrointestinal toxicity of colchicine analogues for the first time based on molecular docking analysis of ligand–protein interactions between the intestinal tight junction protein ZO-1 and dozens of small-molecule compounds. Both the binding energy and interaction mode were investigated to reveal the structure–toxicity relationship and the mechanism of action of COL analogues. Chemical modifications on ring A or ring C, especially the demethylation, greatly impact ZO-1 related activities of COL, which would be significantly attenuated by metabolic transformation via phase I and/or phase II reaction, therefore providing an effective impact detoxication pathway for this naturally occurring drug.

## Figures and Tables

**Table 1:** The ligands with definite ZO-1 related activities used as references for docking analysis *.

Type V—Injury Protector		Type VI—Injury-Causing Agent	
No.	Name	No.	Name
**51**	Glutamine	**59**	Chlorpromazine
**52**	Rebamipide	**60**	Clopidogrel
**53**	L-arginine	**61**	Menthol
**54**	Dexmedetomidine	**62**	Methotrexate
**55**	Quercetin	**63**	Olmesartan
**56**	Ulinastatin	**64**	Bisphenol A
**57**	Emodin	**65**	Rotenone
**58**	Curcumin		

* Chemical structures of these ligands are shown in Supplementary Materials.

**Table 2:** Molecular docking statistics for interaction between ZO-1 and COL with its metabolites.

No.	Interaction Force	BE (-kcal/mol)	Hydrogen Bonding Site
**1** **COL**	hydrophobic interaction electrostatic attraction van der Waals forces	51.17	C7-amido C9-carbonyl
**2** **3-DMC**	hydrophobic interaction electrostatic attraction	35.34	C1-methoxyl C3-hydroxyl
**3** **3-DMC-*O*-sulfate**	electrostatic attraction salt bridge	31.23	C7-amido C3-sulfo group
**4** **3-DMC-O-glucuronide**	hydrophobic interaction electrostatic attraction van der Waals forces salt bridge	34.72	C3-glucuronyl
**6** **2-DMC**	electrostatic attraction van der Waals forces	32.23	C7-amido
**7** **2-DMC-*O*-sulfate**	hydrophobic interaction electrostatic attraction pi-cation	34.88	C9-carbonyl C2-sulfo group
**8** **2-DMC-O-glucuronide**	electrostatic attraction	35.45	C2-glucuronyl
**18** **Glutathione conjugate**	electrostatic attraction van der Waals forces salt bridge	38.12	C9-SG
**31** **10-DMC**	salt bridge	38.64	C7-amido

## Data Availability

The details of the data supporting the report results in this research were included in the paper and Supplementary Materials.

## Supplementary Materials

The following supporting information can be downloaded at: https://www.mdpi.com/article/10.3390/molecules27061797/s1, Table S1: Binding energy of ZO-1 interacting with ligands. Table S2: Reference sources for all colchicine compounds. Figure S1: Intestinal protective drug structure. Figure S2: Intestinal injury drug structure. Figure S3: Interaction diagram of colchicine derivatives and ZO-1.
