# Supplementary material for: A New Insight into Toxicity of Colchicine Analogues by Molecular Docking Analysis Based on Intestinal Tight Junction Protein ZO-1
Source: Molecules. 2022 Mar 9;27(6):1797. doi: 10.3390/molecules27061797 (PMC8955668; doi:10.3390/molecules27061797)
Supplement: Supplementary file 1 [file molecules-27-01797-s001.zip › molecules-1618948-supplementary.pdf]

molecules

MDPI

Table S1. Binding energy of ZO-1 interacting with ligands

NO	Binding energy (-kcal/mol)	NO	Binding energy (-kcal/mol)	NO	Binding energy (-kcal/mol)	NO	Binding energy (-kcal/mol)
**1**	51.17	**21**	37.22	**41**	58.54	**61**	49.83
**2**	35.34	**22**	42.07	**42**	53.34	**62**	52.38
**3**	31.24	**23**	40.64	**43**	54.56	**63**	49.27
**4**	34.72	**24**	58.56	**44**	55.62	**64**	48.59
**5**	50.05	**25**	39.91	**45**	53.10	**65**	54.28
**6**	32.23	**26**	45.24	**46**	43.94		
**7**	34.88	**27**	42.21	**47**	40.81		
**8**	35.45	**28**	61.79	**48**	36.79		
**9**	38.65	**29**	56.02	**49**	46.97		
**10**	45.67	**30**	55.36	**50**	42.09		
**11**	43.25	**31**	38.74	**51**	25.06		
**12**	44.40	**32**	31.40	**52**	34.68		
**13**	44.47	**33**	36.95	**53**	29.15		
**14**	55.62	**34**	32.07	**54**	35.89		
**15**	53.85	**35**	35.93	**55**	30.54		
**16**	56.72	**36**	50.50	**56**	41.32		
**17**	38.56	**37**	54.68	**57**	35.66		
**18**	38.12	**38**	52.10	**58**	39.73		
**19**	38.90	**39**	52.18	**59**	57.70		
**20**	42.30	**40**	48.84	**60**	48.18		

Table S2. Reference sources for all colchicine compounds. *

NO	Reference source
1	Chemistry of colchicine (1991)
2	Identification of Novel Metabolites of Colchicine in Rat Bile Facilitated by Enhanced Online Radiometric Detection (2008)
	The Metabolism of Colchicine by Mammalian Liver Microsomes (1974)
3	Identification of Novel Metabolites of Colchicine in Rat Bile Facilitated by Enhanced Online Radiometric Detection (2008)
	The Metabolism of Colchicine by Mammalian Liver Microsomes (1974)
4	Identification of Novel Metabolites of Colchicine in Rat Bile Facilitated by Enhanced Online Radiometric Detection (2008)
	The Metabolism of Colchicine by Mammalian Liver Microsomes (1974)
5	Therapeutic Use of Colchicine and its Derivatives: A Patent Review (2016)
6	Identification of Novel Metabolites of Colchicine in Rat Bile Facilitated by Enhanced Online Radiometric Detection (2008)
	The Metabolism of Colchicine by Mammalian Liver Microsomes (1974)
7	Identification of Novel Metabolites of Colchicine in Rat Bile Facilitated by Enhanced Online Radiometric Detection (2008)
	The Metabolism of Colchicine by Mammalian Liver Microsomes (1974)
8	Identification of Novel Metabolites of Colchicine in Rat Bile Facilitated by Enhanced Online Radiometric Detection (2008)
	The Metabolism of Colchicine by Mammalian Liver Microsomes (1974)
9	A new alkaloids from Gloriosa sup erba (1993)
10	Substances from the plants of the subfamily Wurmbaeoideae and their derivatives. LVIII. The constitution of cornigerine (1964)
11	Antitumor agents. 185. Synthesis and biological evaluation of tridemethylthiocolchicine analogues as novel topoisomerase II inhibitors (1998)

12	Substances from the plants subfamily wurmbaeoideae and their derivatives
13	Substances from the plants of the subfamily Wurmbaeoideae and their derivatives. LVIII. The constitution of cornigerine
14	Antiproliferative Activity and Molecular Docking of Novel Double-Modified Colchicine Derivatives (2018)
15	Antiproliferative Activity and Molecular Docking of Novel Double-Modified Colchicine Derivatives (2018)
16	Antiproliferative Activity and Molecular Docking of Novel Double-Modified Colchicine Derivatives (2018)
17	Electrophilicities and protein covalent binding of demethylation metabolites of colchicine (2016)
18	Identification of Novel Metabolites of Colchicine in Rat Bile Facilitated by Enhanced Online Radiometric Detection (2008)
	The Metabolism of Colchicine by Mammalian Liver Microsomes (1974)
19	Therapeutic Use of Colchicine and its Derivatives: A Patent Review (2016)
20	Colchicine. Some Reactions of Ring C1 (1952)
21	Not yet synthesized, based on literature extensions
22	Synthesis and Antiproliferative Screening Of Novel Analogs of Regioselectively Demethylated Colchicine and Thiocolchicine (2020)
23	Not yet synthesized, based on literature extensions
24	Synthesis and biological evaluation of novel carbazole-rhodanine conjugates as topoisomerase II inhibitors (1998)
25	Synthesis and Antiproliferative Screening Of Novel Analogs of Regioselectively Demethylated Colchicine and Thiocolchicine (2020)
26	Synthesis and Antiproliferative Screening Of Novel Analogs of Regioselectively Demethylated Colchicine and Thiocolchicine (2020)
27	Not yet synthesized, based on literature extensions
28	Synthesis and biological evaluation of novel carbazole-rhodanine conjugates as topoisomerase II inhibitors (1998)
29	Therapeutic Use of Colchicine and its Derivatives: A Patent Review (2016)
30	Therapeutic Use of Colchicine and its Derivatives: A Patent Review (2016)
31	Identification of Novel Metabolites of Colchicine in Rat Bile Facilitated by Enhanced Online Radiometric Detection (2008)
	The Metabolism of Colchicine by Mammalian Liver Microsomes (1974)
32	Synthesis and Biological Evaluation of Tridemethylthiocolchicine Analogues as Novel Topoisomerase II Inhibitors (1998)
	ZINC database and ChEMBL database search
33	A simplified isolation procedure for azadirachtin (1987)
34	A simplified isolation procedure for azadirachtin (1987)
35	A simplified isolation procedure for azadirachtin (1987)
36	Alkaloids and phenolics of w urmbea and burchardia species (1987)
	Isolation of new copmounds from Colchicum autumnale (1950)
37	Colchicine Glycorandomization Influences Cytotoxicity and Mechanism of Action (2006)
38	Synthesis, Antiproliferative Activity and Molecular Docking Studies of Novel Doubly Modified Colchicine Amides and Sulfonamides as Anticancer Agents (2020)
39	Synthesis, Antiproliferative Activity and Molecular Docking Studies of Novel Doubly Modified Colchicine Amides and Sulfonamides as Anticancer Agents (2020)
40	Inhibition of the Glycine Receptor alpha 3 Function by Colchicine (2020)
41	Arylureas derived from colchicine: Enhancement of colchicine oncogene downregulation activity (2018)
42	Synthesis, Antiproliferative Activity and Molecular Docking Studies of Novel Doubly Modified Colchicine Amides and Sulfonamides as Anticancer Agents (2020)

43	Synthesis, Antiproliferative Activity and Molecular Docking Studies of Novel Doubly Modified Colchicine Amides and Sulfonamides as Anticancer Agents (2020)
44	New Series of Double-Modified Colchicine Derivatives: Synthesis, Cytotoxic Effect and Molecular Docking (2020)
45	Synthesis, Antiproliferative Activity and Molecular Docking Studies of Novel Doubly Modified Colchicine Amides and Sulfonamides as Anticancer Agents (2020)
46	Semkyntheses, X-Ray Crystal Structures and TubulinBinding Properties of 7-Oxodeacetamidocolchicine and 7-0xodeacetamidoisocolchicin
47	Synthesis and Characterization of a Cobalamin-Colchicine Conjugate as a Novel Tumor-Targeted Cytotoxin (2004)
48	Total Synthesis of (−)-Colchicine via a Rh-Triggered Cycloaddition Cascade
49	Pharmaceutical composition for the therapy of diseases caused by highly proliferating cells (2016)
50	New Series of Double-Modified Colchicine Derivatives: Synthesis, Cytotoxic Effect and Molecular Docking (2020)
	Biological Effects of Modified Colchicines. Improved Preparation of 2-Demet hylcolchicine, 3-Demethylcolchicine, and (+)-Colchicine and Reassignment of the Position of the Double Bond in Dehydro-7-deacetamidocolchicines (1981)

* Compounds above include colchicine and its metabolites, derivatives, synthetic intermediates, colchicine analogs and so on.

Figure S1. Intestinal protective drug structure

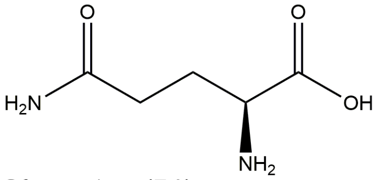

Glutamine (**51**)

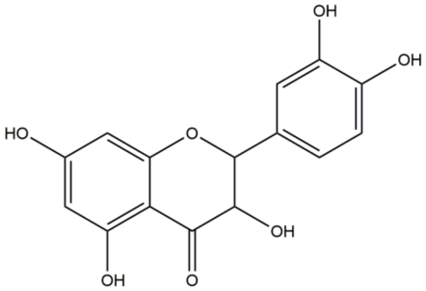

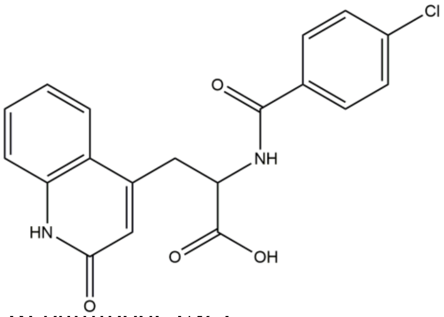

[illegible]

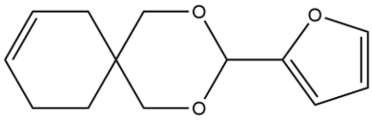

Ulinastatin (**56**)

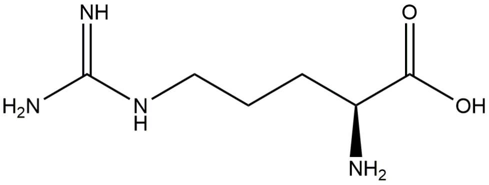

L-arginine (**53**)

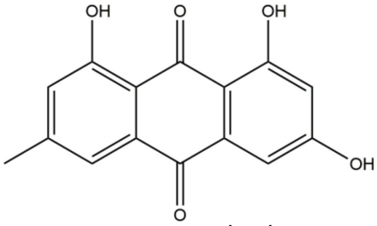

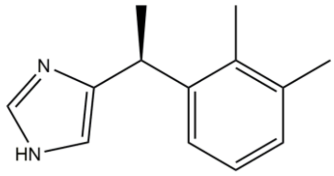

Dexmedetomidine (**54**)

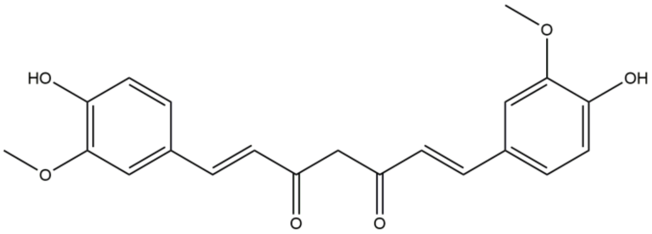

Curcumin (**58**)

Figure S2. Intestinal injury drug structure

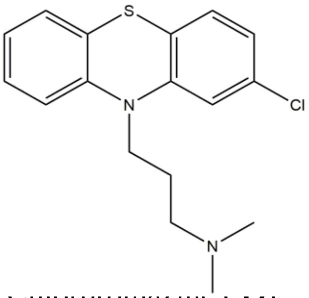

Chlorpromazine ([illegible])

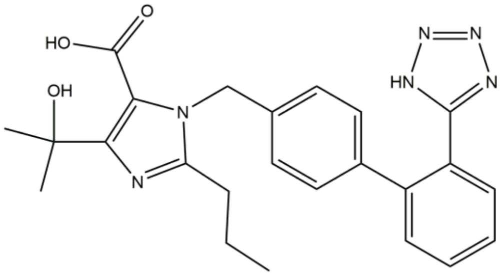

Olmesartan (**63**)

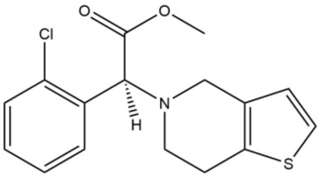

Clopidogrel (**60**)

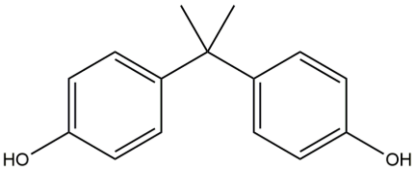

Bisphenol A (**64**)

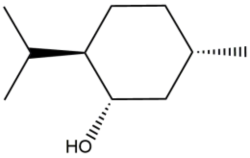

Menthol (**61**)

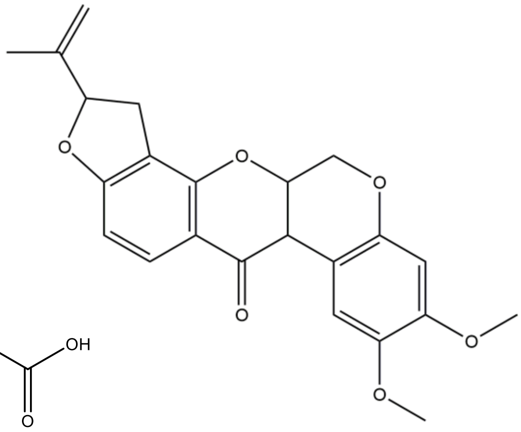

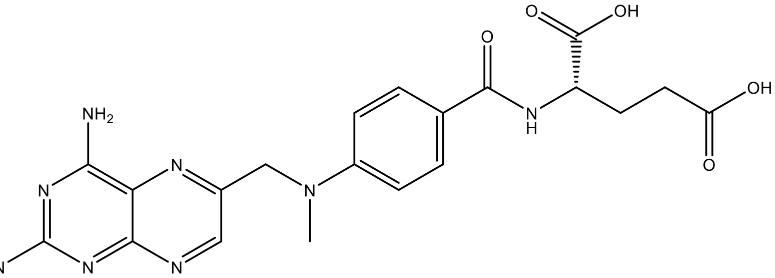

Methotrexate (**62**)

Figure S3. Interaction diagram of colchicine derivatives and ZO-1

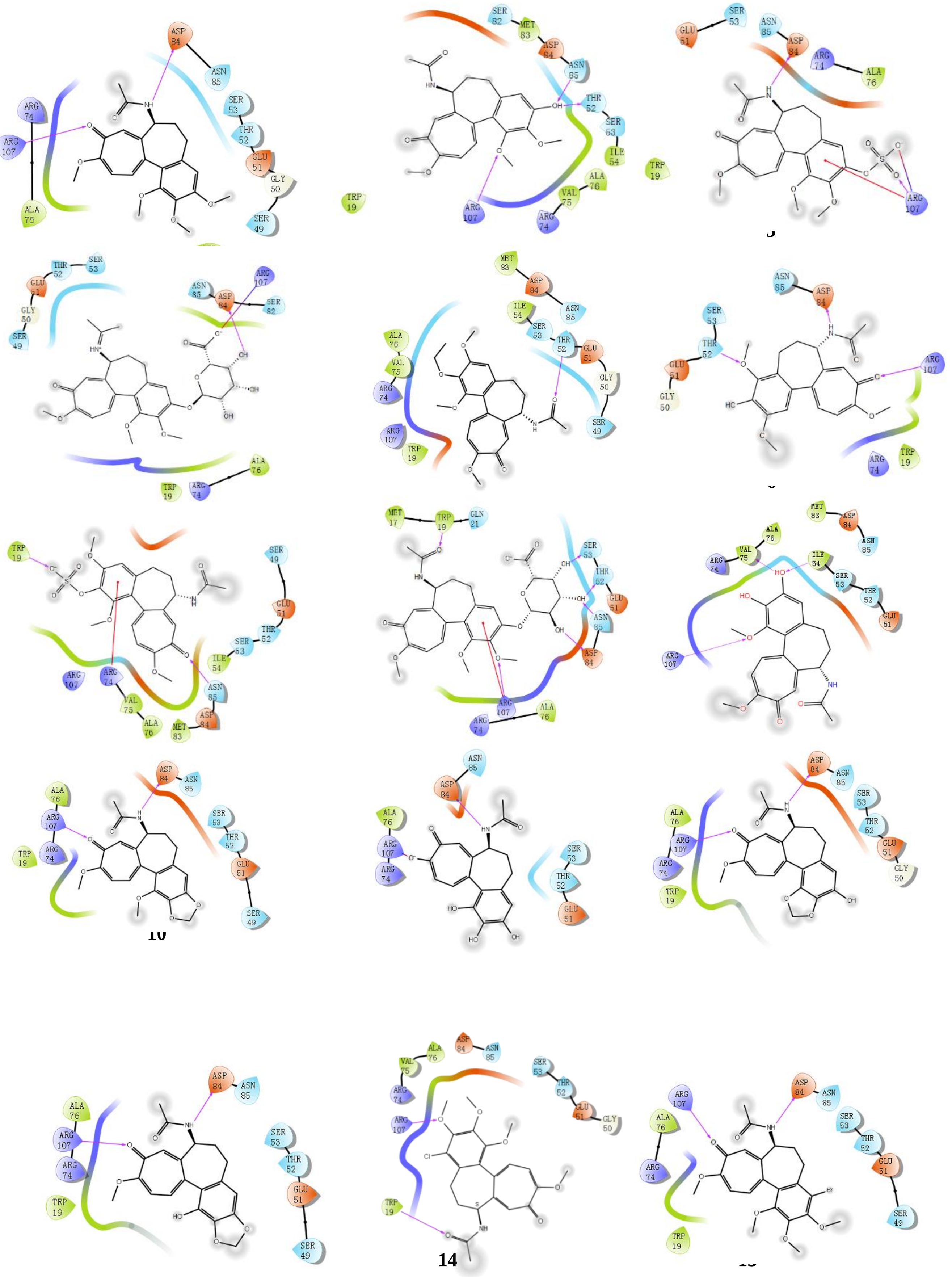

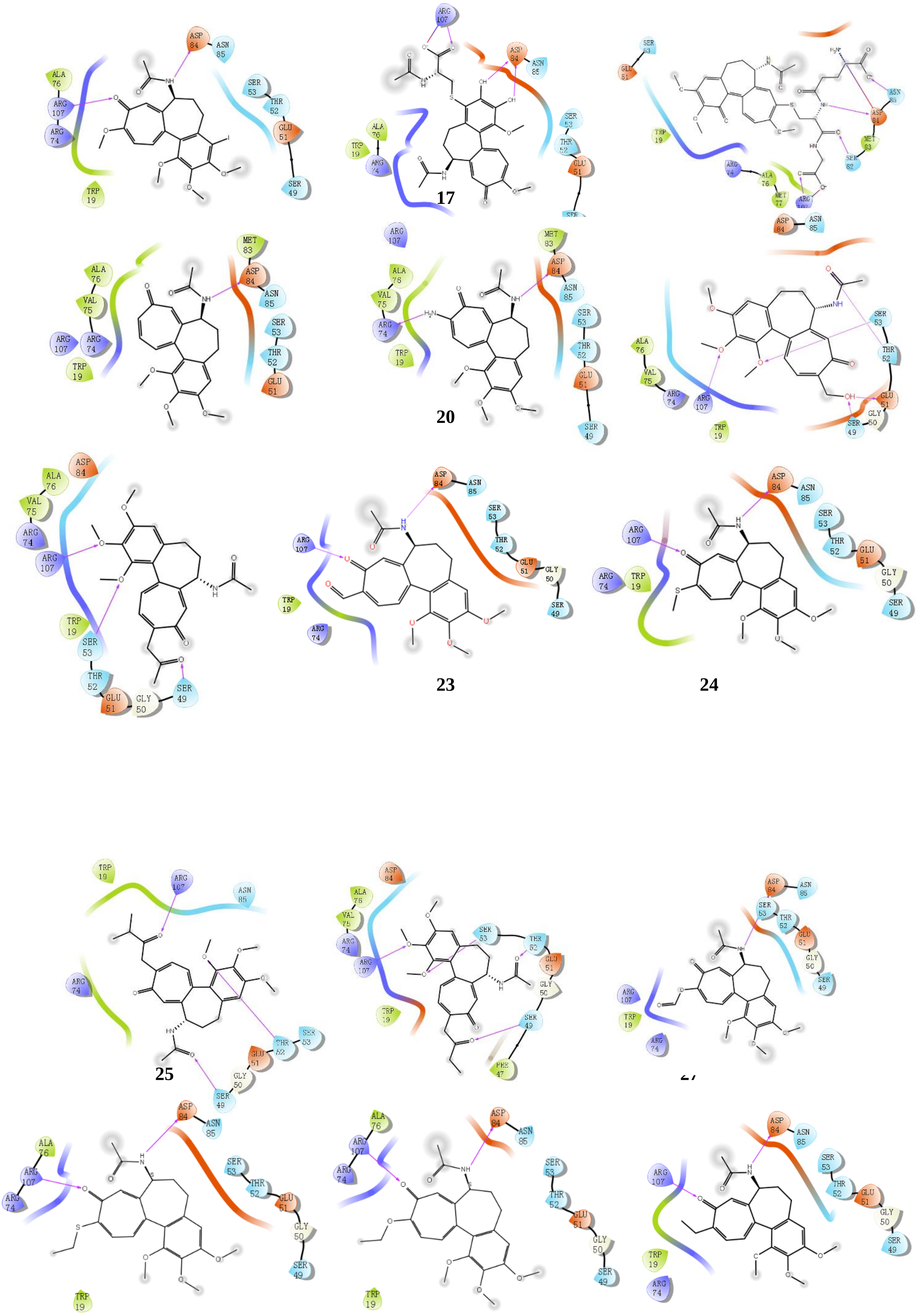
17
20
23
24
25

28

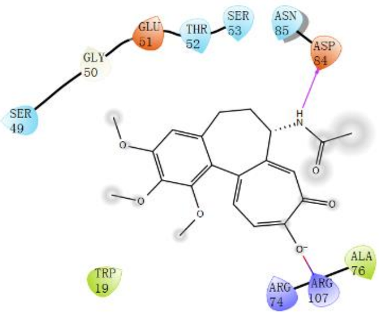

29

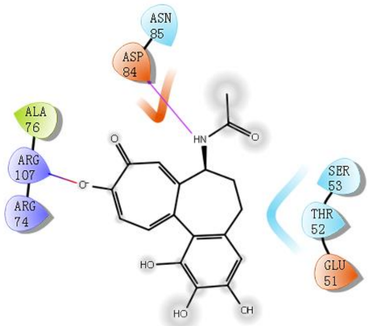

30

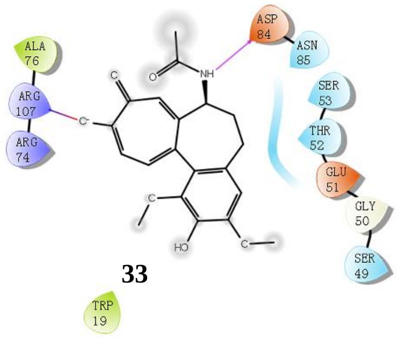

33

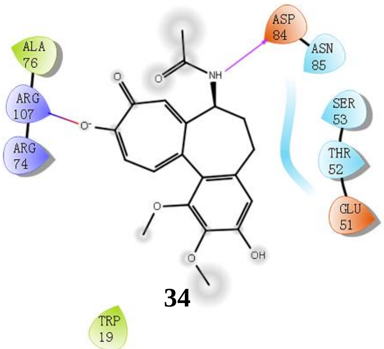

34

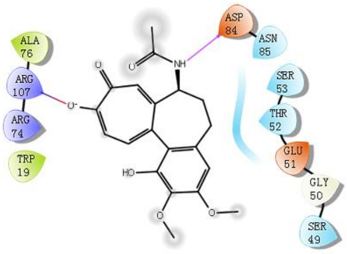

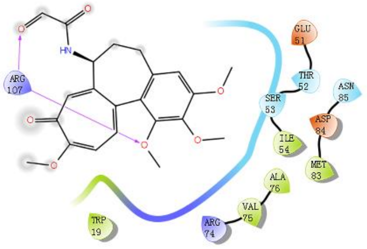

36

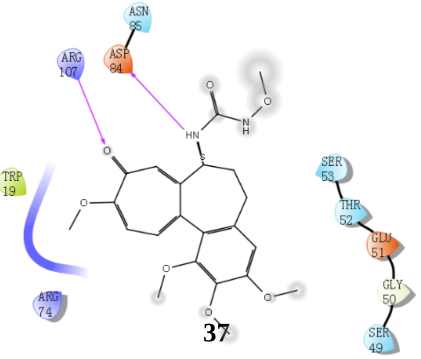

37

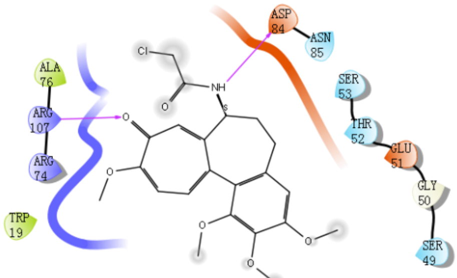

38

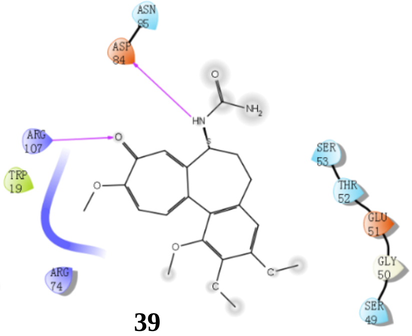

39

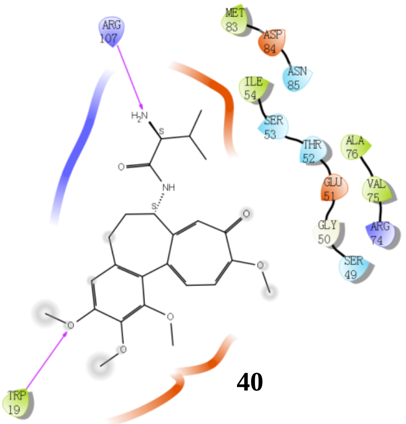

40

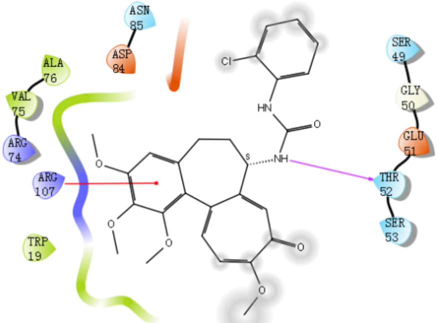

41

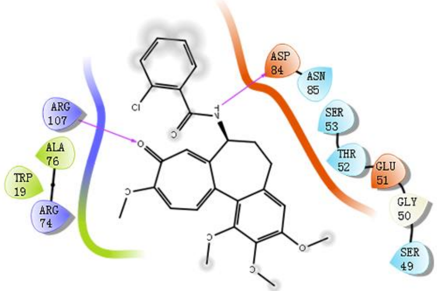

42

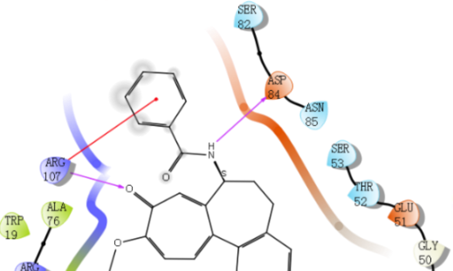

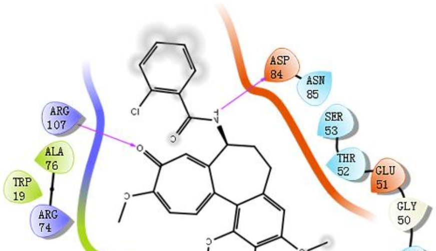

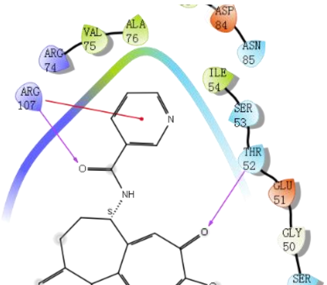

43 **44** **45**

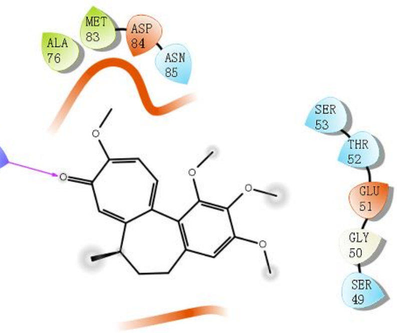
ALA 76
MET 83
ASP 84
ASN 85
SER 53
THR 52
GLU 51
GLY 50
SER 49

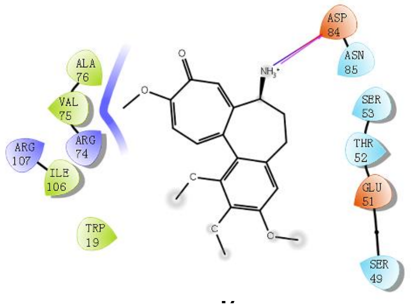
ALA 76
VAL 75
ARG 74
ARG 107
ILE 106
TRP 19
ASP 84
ASN 85
SER 53
THR 52
GLU 51
SER 49

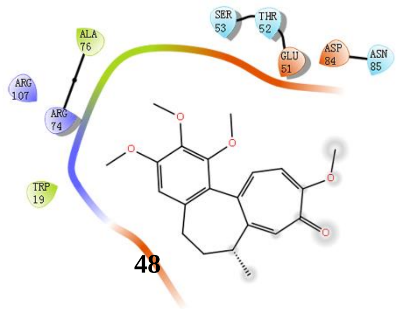
ALA 76
ARG 107
ARG 74
TRP 19
SER 53
THR 52
GLU 51
ASP 84
ASN 85

48

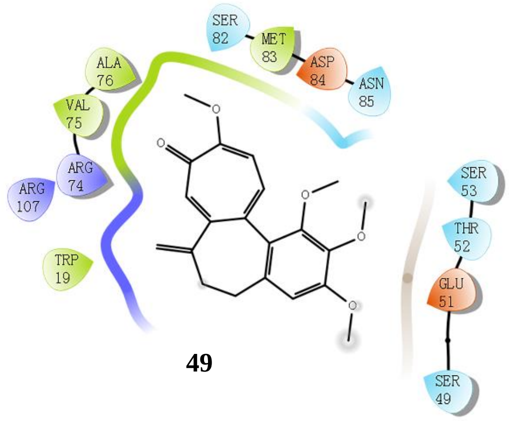
SER 82
MET 83
ASP 84
ASN 85
ALA 76
VAL 75
ARG 74
ARG 107
TRP 19
SER 53
THR 52
GLU 51
SER 49

49

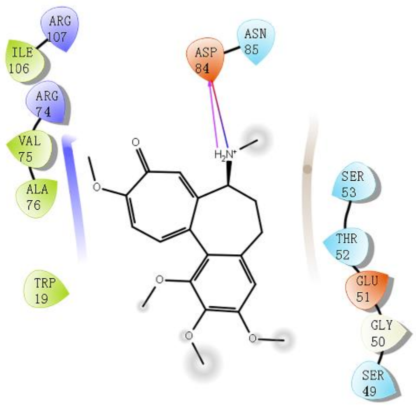
ARG 107
ILE 106
ARG 74
VAL 75
ALA 76
TRP 19
ASP 84
ASN 85
SER 53
THR 52
GLU 51
GLY 50
SER 49
